# Supplementary material for: Enhancing the Properties of Polyvinyl Alcohol Films by Blending with Corn Stover-Derived Cellulose Nanocrystals and Beeswax
Source: Polymers (Basel). 2023 Nov 4;15(21):4321. doi: 10.3390/polym15214321 (PMC10648525; doi:10.3390/polym15214321)
Supplement: Supplementary file 1 [file polymers-15-04321-s001.zip › polymers-2689267-supplementary.pdf]

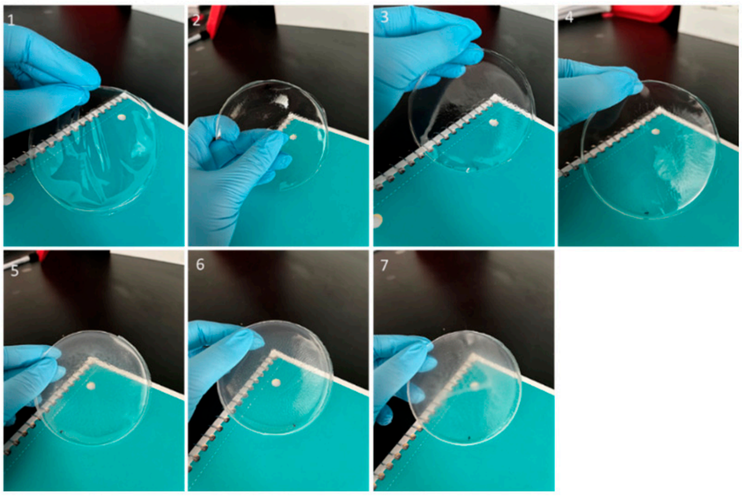

Figure S1. Images of coating films. 1: 3% (w/v) Polyvinyl alcohols (PVAs), 2: 3% (w/v) PVAs + 0.075% (w/v) cellulose nanocrystals (CNCs), 3: 3% (w/v) PVAs + 0.15% (w/v) CNCs , 4: 3% (w/v) PVAs + 0.3% (w/v) CNCs, 5: 3% (w/v) PVAs + 0.075% (w/v) CNCs + 1.5% (w/v) Beeswax, 6: 3% (w/v) PVAs + 0.15% (w/v) CNCs + 1.5% (w/v) Beeswax, and 7: 3% (w/v) PVAs + 0.3% (w/v) CNCs + 1.5% (w/v) Beeswax.
